# End-User Perspectives on Using Quantitative Real-Time PCR and Genomic Sequencing in the Field

**DOI:** 10.3390/tropicalmed7010006

**Published:** 2022-01-05

**Authors:** Kyle Parker, Jonathan Forman, George Bonheyo, Brittany Knight, Rachel Bartholomew, Richard Ozanich, Kenneth B. Yeh

**Affiliations:** 1 MRIGlobal, 425 Dr. Martin Luther King Jr. Blvd., Kansas City, MO 64110-2241, USA; bknight@MRIGLOBAL.ORG (B.K.); kyeh@mriglobal.org (K.B.Y.); 2 Pacific Northwest National Laboratory, 902 Battelle Blvd., Richland, WA 99354, USA; Jonathan.Forman@pnnl.gov (J.F.); george.bonheyo@pnnl.gov (G.B.); Rachel.Bartholomew@pnnl.gov (R.B.); Richard.Ozanich@pnnl.gov (R.O.)

**Keywords:** biodefense, qPCR, sequencing, field laboratories, first responders, military, defense

## Abstract

Quantitative real-time PCR and genomic sequencing have become mainstays for performing molecular detection of biological threat agents in the field. There are notional assessments of the benefits, disadvantages, and challenges that each of these technologies offers according to findings in the literature. However, direct comparison between these two technologies in the context of field-forward operations is lacking. Most market surveys, whether published in print form or provided online, are directed to product manufacturers who can address their respective specifications and operations. One method for comparing these technologies is surveying end-users who are best suited for discussing operational capabilities, as they have hands-on experience with state-of-the-art molecular detection platforms and protocols. These end-users include operators in military defense and first response, as well as various research scientists in the public sector such as government and service laboratories, private sector, and civil society such as academia and nonprofit organizations performing method development and executing these protocols in the field. Our objective was to initiate a survey specific to end-users and their feedback. We developed a questionnaire that asked respondents to (1) determine what technologies they currently use, (2) identify the settings where the technologies are used, whether lab-based or field-forward, and (3) rate the technologies according to a set list of criteria. Of particular interest are assessments of sensitivity, specificity, reproducibility, scalability, portability, and discovery power. This article summarizes the findings from the end-user perspective, highlighting technical and operational challenges.

## 1. Introduction

Military, defense, and intergovernmental entities such as the United Nations, which coordinated missions to oversee disarmament in Iraq, frequently deploy field transportable and mobile laboratories [1,2] to address situations involving biological weapons of mass destruction (WMD). The modalities of operation for these missions are often expeditionary and include countering biological WMD and bioterrorism while maintaining force protection to ensure preparedness and readiness. Although the Biological Weapons Convention does not have an implementing body, the United Nations Secretary General’s Mechanism may exercise future investigations of alleged use of biological weapons with support from a network of laboratories [3]. First responders including fire, rescue, and law enforcement professionals are tasked with countering and triaging unknown threats to public safety. Operating within dynamic and challenging environments also requires the ability to multitask and be proficient at sampling and detection using a variety of equipment and test kits. Each situation is also challenged with the need for rapid turnaround time for results, multiple reporting structures and changing priorities. Critical decision making is essential for maintaining posture and readiness, and implications have far-reaching outcomes for safety and security of the operators and those they protect.

Identifying biothreats in the field quickly and accurately requires technology that is robust, reliable, and culture-independent. Molecular detection methods including real-time quantitative polymerase chain reaction (qPCR) and next-generation sequencing (NGS) are widely used for an array of biological threat applications. Currently, qPCR is considered the “gold standard” in molecular detection, as well as the basis for most genomic sequencing applications. The typical concept of operation requires a sample preparation step, after which the resulting nucleic acids can then be run by qPCR and genomic sequencing in parallel. qPCR methods are typically employed when there is a priori knowledge of expected target detection or when there is the need for presence/absence confirmation of an intended target. Next-generation sequencing methods are typically used to query samples for metagenomic analysis. The output from these sequencing runs often provides highly complex data for analysis, relative to data outputs from qPCR runs which may be as simple as a “yes/no” detection result. 

Recent trends, as evidenced by COVID-19 and outbreaks of the Ebola virus, continue to emphasize the need for accurate, rapid diagnostic tests that can be performed outside traditional laboratory settings, especially at the point of care [4,5]. The COVID-19 tests with FDA emergency use authorizations (EUA) for in vitro diagnostic devices include molecular-based detection methods (qPCR and genomic sequencing) and antigen-based immunoassays, which are now commercially available for point-of-care and home-based testing [6]. Genomic sequencing technologies are currently termed first, second, and third generation [7]. Sanger sequencing, which only sequences a single DNA nucleotide at a time using capillary electrophoresis, is widely considered first generation. The second and third generations are often referred to as next-generation sequencing (NGS) [8]. Second generation refers to template amplification prior to sequencing and includes Ion Torrent, Pacific Biosciences, and Illumina platforms [9]. Third-generation sequencing such as Oxford Nanopore does not require a template amplification step and offers point-of-care sequencing capability with small portable devices and automated sample preparation capabilities [10,11]. Related to biosurveillance, which would also cover bioforensics and clinical diagnostics, NGS offers an enhanced complement to molecular methods for detection, identification, and characterization of biological threats [12]. High accuracy has been demonstrated for field-portable nanopore sequencing devices for COVID-19 diagnosis [5]. The regulatory approvals for these and related devices reflect their product maturity over newer state-of-art-technology when testing clinical samples.

In comparison with qPCR and immunoassays that provide detection and possible quantification of specified targets, NGS methods have limited quantitative capability, but provide genomic data that may be used both to detect specified targets and to identify and characterize unknown biological entities. In its current state, concepts of operation are applicable to these applications, especially those unknown emerging infectious diseases in addition to environmental sampling [13,14]. The power of NGS offers a greater depth of knowledge with sequencing of all nucleic acids in a sample: a less-biased approach and contrary to traditional a priori detection of biological agents.

Molecular detection technologies for biological threats in the field and laboratory are well documented [15,16,17]. Yet, while various methods and case histories for deploying field laboratories have been presented [1,18,19,20], few peer-reviewed publications specify commercially available biodetection systems [21,22,23]. Since the biodefense market and enterprise has evolved following the 2001 anthrax attacks, several marketing references surveying various biological threat detection technologies have been published and presented online [24,25]. These references include published market surveys of biological detectors and related technologies aimed at audiences of first responders, research and development, and the scientific community. Various analyses have been developed from product manufacturer specifications to show theoretical use cases of a given technology in a laboratory-centric rather than an end-user operation. While current surveys have evolved into interactive formats, which make choosing and comparing various products convenient, the lack of end-user perspective makes the references more textbook in utility than real-world.

Our online search for open-source market surveys (including a Google Scholar citation if available) is summarized in Table 1. While these surveys are extensive and provide the manufacturers’ technical assessments of their products, there were only two designed with input from end-users [20,26]. Qualitative metrics, such as real-world experience on testing samples according to sensitivity, specificity, accuracy, and ruggedness are valuable for end-users making assessments on appropriate technologies to incorporate into their field operations. End-user experience is important to validate actual field use and to balance inherent bias from manufacturer provided feedback in market surveys. Here, we focus on gaining perspectives on using qPCR and sequencing from end-users in the field, which offer important and realistic ease of use on the basis their hands-on experience. 

## 2. Methods/Background, Design Distribution of Questionnaire

For this manuscript, we constructed a questionnaire survey that asked respondents to evaluate and rate operational discriminators for qPCR and sequencing technologies. We invited known end-users with previous experience with these applications to provide their insight into the benefits and limitations of current and emerging technologies. To limit bias, we did not solicit input from respondents whose current positions involve the development, marketing, or sale of qPCR and sequencing instruments or consumables. Previously, Yeh et al. discussed a notional qualitative comparison of qPCR and sequencing technologies, according to literature searches and panel feedback [18]. The qualitative comparison included the following evaluation metrics: sensitivity, specificity, reproducibility, ease of use, time to detection, low cost per test, discovery power, scalability, portability, and ruggedness. For this manuscript, we sought feedback from end-users on their actual experience with qPCR and sequencing technologies, both in the field and in laboratory settings. To gain this feedback, we designed a survey that asked respondents to evaluate the technologies using the evaluation criteria listed above. We defined each of the evaluation criteria as described in Table 2 below. The questionnaire was designed to determine which technologies the respondent was familiar with (qPCR, sequencing, or both) and then rate them individually on a scale of 1 to 5. A score of 5 indicates that the technology meets the criteria defined for each metric; a score of 1 indicates that the technology falls short of the defined metric criteria. All evaluation criteria were weighted evenly. To provide additional information, respondents were asked to list what types of instrumentation they were familiar with and in what settings they typically deployed these technologies. We categorized the instruments into groups, which are described in Table 3, on the basis of a high-level similarity in overall function or technology. We also included a category for “point-of-use” platforms, defined as those platforms that can be used in nontraditional laboratory settings. Finally, at the end of the survey, respondents were asked to provide information on their current position, area of expertise, and education. 

Candidate respondents were solicited by email from the author’s network of contacts, spanning diverse fields such as animal health, biosurveillance, clinical diagnostics, human forensics, and infectious diseases, including individuals from several countries. Input was sought from end-users with a diversity of experience, from laboratory technicians to principal investigators. The survey results were compiled and analyzed to determine trends amongst the responses. 

## 3. Results

### 3.1. Survey Respondents

Participants were asked to provide some basic background information to correlate with their responses. These were their highest level of education, their current position, and for what applications they use qPCR and/or sequencing technologies. 

Although limited in the number of respondents, these results provide a representation of valuable insights from personnel that routinely use the instruments, as well as some of the decision makers who are responsible for identifying instruments for purchase and integration into process workflows. Regardless of role, whether they operate the instruments directly or if they manage a team that operates the instruments, the respondents would all understand performance metrics that would impact their workflows.

Lastly, respondents were asked to identify what applications they are currently using or have used these technologies for.

### 3.2. User Experience

Prior to asking respondents to perform a qualitative assessment of qPCR and sequencing technologies, we requested background information on their experiences using these methods. In the first question, participants were asked to indicate if they have experience using qPCR or sequencing technologies or both. All 18 respondents (100%) had experience with qPCR, while 15 (80%) had experience with some sequencing technologies. This result was not surprising, as end-point PCR amplification is a routine molecular method and inherent to most sequencing methods; however, it is possible that someone could be proficient with sequencing techniques and not with qPCR.

As a follow-up, participants were asked to indicate what type of instruments that they are familiar with. The respondents indicated familiarity across a wide variety of platforms, from high-throughput instruments to handheld, portable devices according to the classifications provided in Table 3. Over half of the respondents said that they had experience with instruments in each category. For sequencing platforms, a majority of the respondents had experience with first-, second-, and third-generation sequencing instruments. 

### 3.3. Qualitative Assessment

**Figure 1:**
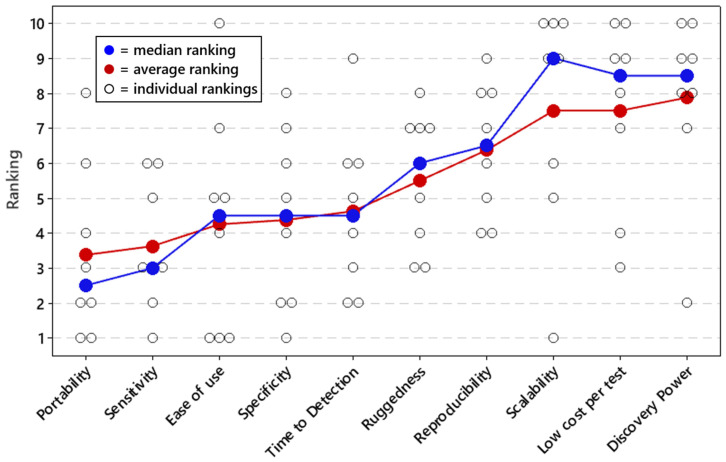
Priorities for field-forward qPCR applications. Respondents ranked all performance metrics on a priority scale of 1 to 10, with 1 being the most important and 10 being the least important. The average and median rankings for each performance metric are provided in Figure 1, as well as each individual response. Average and median rankings are based on responses from n = 8 respondents.

**Figure 2:**
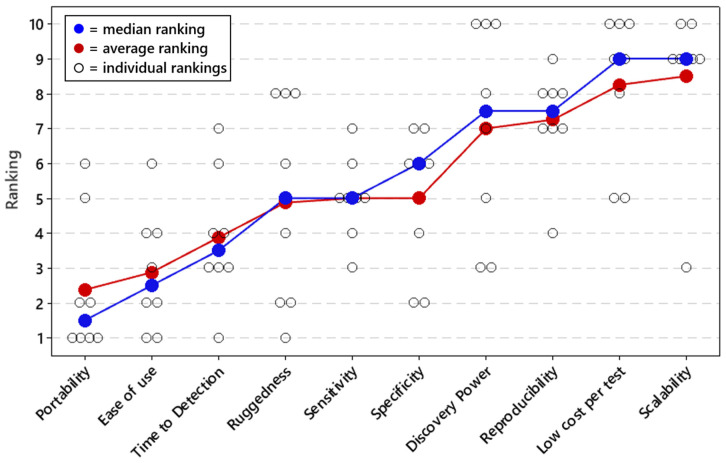
Priorities for field-forward sequencing applications. Respondents ranked all performance metrics on a priority scale of 1 to 10, with 1 being the most important metric and 10 being the least important. The average and median rankings for each performance metric are provided in Figure 2, as well as each individual response. Average and median rankings are based on responses from n = 8 respondents.

Respondents were asked to rank the importance of the 10 performance metrics for field-forward applications of qPCR and sequencing (Figure 1 and Figure 2). According to both median and average ranking, portability was the highest ranked metric for both qPCR and sequencing applications. After that, ease of use and time to detection were the next most important metrics for sequencing. For qPCR, sensitivity was the second most important metric, followed by ease of use, specificity, and time to detection. These three metrics all had similar median and average rankings. 

## 4. Challenges in Field-Forward Settings

**Figure 3:**
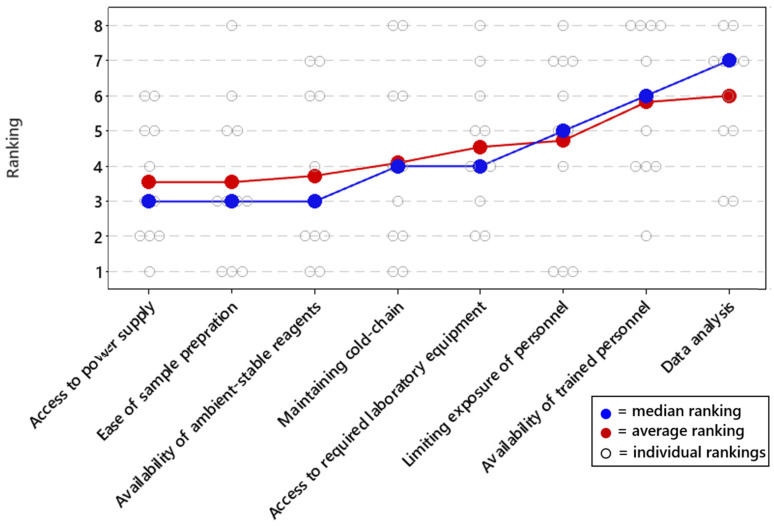
Challenges for field-forward biodetection applications. Respondents ranked all challenges on a priority scale of 1 to 8, with 1 being the most important and 8 being the least important issue to overcome. The average and median rankings for each performance metric are provided in Figure 3, as well as each individual response. Average and median rankings are based on responses from n = 11 respondents.

Next, respondents were asked to provide feedback on the overall challenges of performing qPCR and sequencing in field-forward settings. First, we asked the respondents to rank common issues with field-forward applications to determine the most common challenges that need to be addressed (Figure 3). Access to power supply, ease of sample preparation, and the availability of ambient-stable reagents were found to be the most important. These three challenges all had similar average and median rankings. 

**Figure 4:**
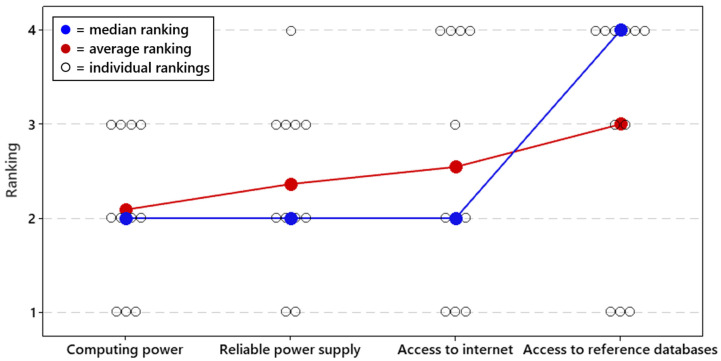
Priorities for field-forward sequencing applications. Respondents ranked all challenges on a priority scale of 1 to 4, with 1 being the most important and 4 being the least important issue to overcome. The average and median rankings for each challenge are provided in Figure 4, along with all individual responses.

Respondents were then asked to specifically address data analysis challenges in the field (Figure 4). Here, the challenges were all evenly ranked, with computing power reliable power supply and access to the internet all having the same median ranking. Access to reference databases had a significantly lower median ranking. 

## 5. Discussion

The survey results confirmed that qPCR and sequencing applications have different benefits and challenges in the field for biosurveillance applications. While qPCR methods are typically low-cost and provide rapid turnaround detection with high sensitivity, sequencing methods are more reliable for discovery power. As these technologies move from laboratory settings to field-forward applications, we observed a convergence of end-user ratings on performance metrics. Both field-forward and portable qPCR and sequencing platforms show a shift toward increasing ease of use, portability, ruggedness, and time to results. On the other hand, both technologies show a decrease in sensitivity and reproducibility when compared to laboratory-based platforms. While the cost to perform sequencing is still high in both the field and the lab, we noted that field-based PCR techniques are more expensive than lab-based technologies, where higher throughput allows for cost savings. The COVID-19 pandemic has also shown there are many scenarios for field-forward deployments such as providing high-throughput testing, implementing rapid diagnostics, and providing access to low resource settings. As part of a whole-government response, civilian and military cooperation deployed assets including mobile laboratories for testing and clinical diagnostics [33]. The increased number of platforms, kits, and methods available for performing point-of-care and home-based PCR tests should lead to a decrease in testing costs. 

The feedback from end-users on the challenges associated with making these technologies amenable to field-forward applications proved to be the most informative aspect of this survey. The respondents provided feedback that clearly demonstrated their priorities and the current challenges that will need to be addressed. For field-forward applications, both portability and ease of use were determined to be amongst the top three priorities for both qPCR and sequencing technologies. It is noteworthy that sensitivity was ranked in the top three for qPCR, whereas turnaround time was similarly ranked for sequencing. Surprisingly, specificity was not listed as a top priority for field-forward sequencing (ranked as number 5 with sensitivity). Specificity is a key concern for the use and interpretation of sequencing data, but the lower ranking may simply reflect an acknowledgement that field-forward sequencing is an emerging capability and a willingness of researchers to work within existing limitations and to sacrifice specificity for other features. As for the challenges to overcome, aside from having a reliable power supply, the top four were all related to sample preparation in the field. The ability to quickly and accurately prepare samples for analysis is an important topic in the conversation of field-forward biodetection methods.

The importance of sample preparation methods was reflected in the feedback received from the respondents. They noted that “access to dedicated, sterile workspaces for preparation of multiple, consecutive samples”, limiting sample exposure and maintaining the cold chain, are some of the current challenges. Similarly, “many field-forward instruments have limited input volume restrictions. Real-world samples may be bulky, complex, and contain inhibitors”, and it can be difficult to process nonliquid, large-volume, and low-titer samples adequately in the field. One respondent noted that field-forward sample preparation components such as nucleic acid extraction and library preparation should not have to rely on typical laboratory infrastructures such as centrifuges and freezers. One response noted that microfluidic systems such as the “BioFire FilmArray and Oxford Nanopore Technologies VolTRAX platforms are promising technologies that offer potential solutions for field-forward applications”. Technologies such as these increase the ease of use for operators by integrating automated sample preparation steps into the device itself, eliminating the need for some laboratory equipment. The challenges noted here offer insight into the needed improvements in detection methods to support the biosurveillance community, highlighting the work so these technologies can meet the challenges in the field. 

While not covered in this survey, other field-forward nucleic acid analysis technologies are available or in development. For example, loop-mediated isothermal amplification (RT-LAMP) [34] and recombinase polymerase amplification (RPA) [35] assays have been deployed in laboratories and remote settings, and the use of CRISPR/CAS for point-of-care applications has also been demonstrated [36]. Technologies demonstrated for COVID-19 screening include RT-LAMP [37], RPA [38], and CRISPR/CAS [39,40] systems. The power of CRISPR-based diagnostics is the potential for developing a priori assays quicker and eventual programmable/on-demand assays. However, the CRISPR assays currently available for detecting COVID-19 are only in a high-throughput format and comparable to qPCR in sensitivity, specificity, and time to result. Field-forward or point-of-need CRISPR tests are in development and not yet widely available. As new methods emerge that enable targeted cell lysis for sequencing, this may reduce the bioinformatics demands of broad metagenomics sequencing approaches. 

## 6. Conclusions

Survey findings reinforce presumptive published performance metrics of qPCR and sequencing technologies with feedback from current end-users. Currently, qPCR and NGS methods are complementary and interdependent, and there are a limited number of field-forward-capable next-generation sequencing options [13,19]. The survey further revealed both methods have associated challenges. The results also provide guidance for future studies on what sample preparation factors to consider when developing field-forward applications and the important challenges to overcome. Ideally, these technologies would increase ease of use and minimize traditional laboratory equipment or infrastructure while maintaining high-performance metrics observed with traditional qPCR and sequencing instruments. As new applications become available and adopted in the field, future assessments should consider these methods while broadening the scope to a larger target audience of end-users. 

## Figures and Tables

**Table 1:** List of field and first responder biodetection marketing references including print and electronic formats available online. Citations counted from 11 September 2021.

Title, (Year Published), and [Reference Number]	Google Scholar Citations (11 September 2021)
An Introduction to Biological Agent Detection Equipment for First Responders (2001) [24]	30
Biological Detectors Market Survey (2007) [25]	4
Chemical, Biological, Radiological Technology Survey (2011) [26]	7
Edgewood Biosensors Test Bed Handheld and Man-portable edition (2013) [27]	1
WMD Detector Selector (2015) [28]	Website only, not available
CBRNE Tech Index (2015) [29]	Website only, not available
Biodetection Technologies for First Responders (2015) [30]	9
Recommendations on the use of diagnostics devices in far-forward military operations (2016) [31]	1
Global CBRN Detector Market Survey (2017) [32]	2

**Table 2:** Evaluation criteria and definitions. Ten criteria were used in our questionnaire.

Criteria	Definition
*Ease of Use*	The ability to be used by operators with limited training.
*Time to Results*	The ability to quickly produce actionable results.
*Sensitivity*	Analytical sensitivity; ability to measure a low number of copies, genomic equivalents, etc.
*Specificity*	Analytical specificity; ability to detect a particular target. Sequencing accuracy
*Reproducibility*	Ability to generate similar results consistently across sequential runs.
*Portability*	Ability to move instrument from one location to another without impacting instrument integrity.
*Ruggedness*	Ability of instrumentation to withstand significant movement, vibrations, environmental impacts.
*Discovery Power*	Ability to detect novel variants, unknown targets
*Scalability*	The number of samples able to be processed simultaneously, low (1–8) to high (96 or greater).
*Low cost per test*	The cost to process a sample, inclusive of reagents.

**Table 3:** Instrument Categories. The real-time PCR instrument types were based on throughput and portability, and the sequencing instruments were based on sequencing platform generation.

Category	Instrument Type	Definition	Example
Real-time PCR	High-throughput instruments	Platforms with 96-well format or greater	ABI 7500 Fast, Bio-Rad CFX96, QuantStudio™
	Lower throughput instruments	Platforms with less than 96-well format	Cepheid GeneXpert
	“Point of Use” instruments	Platforms with potential use in doctor’s office, clinic, etc.	Abbott ID Now, BioFire Film Array
	Ruggedized instruments	Platforms designed to MIL-810 STD	Idaho Technologies Razor, RAPID
	Hand-held, portable instruments	Platforms used in field-forward or mobile laboratory	Biomeme Franklin
Sequencing	Capillary electrophoresis platforms	First generation sequencing platforms	Sanger Sequencing
	Next-generation sequencing platforms	Second generation sequencing platforms	Illumina MiSeq, 454, Ion Torrent
	Nanopore sequencing platforms	Third generation sequencing platforms	Oxford Nanopore Technologies Prometheon, GridION, MinION
	“Point of Use” platforms	Platforms with consolidated function, consummables	Oxford Nanopore Technologies MK1C, Illumina iSeq

## Data Availability

Not applicable.
